# Age‐Related Structural–Functional Discrepancy in Muscle Indicators Among Rural Korean Older Women

**DOI:** 10.1155/jare/5833371

**Published:** 2026-06-28

**Authors:** Jaeyong Park, Youngju Song

**Affiliations:** ^1^ Department of Fitness Rehabilitation, Sunmoon University, Asan, Republic of Korea, sunmoon.ac.kr

**Keywords:** aging, five-times sit-to-stand test, muscle function, rural women, skeletal muscle index

## Abstract

**Background:**

Age‐related loss of muscle mass and function contributes to mobility limitations, loss of independence, and other adverse health outcomes. Structural and functional indicators characterize muscle health but may not change in parallel with advancing age. Evidence from rural populations remains limited. This study aimed to examine age‐related differences in structural and functional muscle indicators among rural community‐dwelling women and to determine whether age‐group differences are greater in functional than in structural indicators.

**Methods:**

This cross‐sectional study included 211 community‐dwelling women aged 50–89 years residing in rural Asan, Korea. Participants were categorized into four age groups (50–59, 60–69, 70–79, and 80–89 years). Structural indicators included skeletal muscle index (SMI), skeletal muscle mass (SMM), thigh circumference (TC), and calf circumference (CF). Functional indicators included handgrip (HG) strength, isometric knee extensor (KE) strength, and the five‐times sit‐to‐stand test (5×STS). Between‐group differences were analyzed using one‐way analysis of variance (ANOVA), and domain‐level differences were further examined using a mixed‐design repeated‐measures ANOVA. To facilitate comparison across indicators, variables were normalized to the 50–59 age group (set to 100).

**Results:**

Structural and functional indicators showed different magnitudes of age‐related change across groups. HG and KE were lower in older groups and showed larger between‐group differences than the structural indicators. In contrast, 5×STS time increased across age groups, from 7.0 ± 1.75 s in the 50–59 group to 15.2 ± 6.60 s in the 80–89 group. Relative SMI decreased to 81.5% of the reference group, whereas the relative index for 5×STS (reciprocal time) decreased to 46.0% (all *p* < 0.001).

**Conclusions:**

Structural and functional muscle indicators showed divergent age‐related patterns among rural community‐dwelling women. The performance‐based 5×STS demonstrated substantially greater relative differences than structural indicators, supporting the inclusion of functional assessments alongside structural measures for age‐stratified evaluation in rural settings.

## 1. Introduction

Age‐related declines in muscle function are a fundamental feature of aging and are strongly associated with mobility limitations, loss of independence, and increased mortality [1–3]. Functional impairment is consistently linked to adverse health outcomes in later life, including increased risk of disability, hospitalization, and institutionalization [4–6]. Early identification and management of functional decline have become important components of healthy aging strategies, and function‐oriented assessment has received increasing attention [7]. Despite this recognition, it remains unclear whether age‐related changes in muscle function follow a consistent pattern across age groups. Although prior studies have reported lower levels of muscle function in older age groups, the magnitude of these differences appears to vary across age groups [8–10].

Functional decline has traditionally been interpreted in conjunction with the loss of muscle mass. Increasing evidence suggests that age‐related patterns of structural and functional indicators do not necessarily coincide. Whereas muscle mass generally shows gradual differences across age groups, muscle function may exhibit disproportionately larger age‐group differences, potentially reflecting neuromuscular alterations, such as impaired coordination, motor unit remodeling, and reduced neural drive [11, 12]. This distinction suggests that structural and functional characteristics may follow different patterns across age groups.

Age‐stratified research indicates that functional performance does not decline uniformly across age groups. Functional indicators, such as gait speed, handgrip (HG) strength, and sit‐to‐stand (STS) performance, are typically lower in older age groups, with these differences tending to be larger at more advanced ages [13, 14].

Unlike structural indicators, such as muscle mass, muscle function is directly linked to daily performance and may better reflect functional status. Evidence indicates that muscle mass, strength, and physical performance contribute differently to mobility limitation: Declines in strength and performance are more closely associated with impairments in tasks, such as walking and rising from a chair, whereas reductions in muscle mass alone may be insufficient to explain functional limitations [15, 16]. HG strength has been widely adopted as a practical biomarker of overall muscle function in older adults [17, 18]. More recent work indicates that muscle‐specific strength measures and performance‐based indicators may provide additional information beyond structural measures in relation to functional performance and mobility in aging populations [19].

Community context represents an important consideration in the evaluation of muscle health in aging populations. Asan is a mixed urban–rural city located in Chungcheongnam‐do, South Korea. According to municipal statistics, the total population of Asan was approximately 379,000 in 2023, including approximately 132,000 women aged 20 years and older, representing 47.7% of the population aged 20 years and older. Rural areas of Asan are characterized by a relatively high proportion of older adults, whose primary economic activities include agriculture and small‐scale manufacturing. Older adults in these areas may have limited access to structured physical activity programs and healthcare services. Physical activity patterns, occupational demands, and access to health resources differ between urban and rural settings, and women residing in rural communities may experience these influences in distinct ways. Urban–rural disparities have been reported in sarcopenia‐related indicators, with meta‐analytic evidence indicating higher prevalence estimates in rural populations [20]. Preservation of functional capacity is particularly critical for women, given its close association with independent living and community participation. However, systematic age‐stratified investigations of muscle function specifically among rural women remain limited. Existing research has often focused on selected age ranges or singular indicators using mean‐based comparisons, rather than directly comparing structural and functional indicators across clearly defined age groups to determine whether their age‐related differences diverge. It therefore remains unclear whether structural and functional indicators exhibit differential patterns across age strata within rural populations. Clarifying this potential structural–functional divergence may refine muscle health assessment and support more precise identification of individuals at elevated risk of functional impairment in rural aging populations.

This study aimed to examine structural and functional muscle indicators across age groups in rural community‐dwelling women. Structural indicators reflect muscle quantity and include measures, such as skeletal muscle mass (SMM), skeletal muscle index (SMI), and limb circumferences, whereas functional indicators—including HG strength [17, 18], isometric knee extensor (KE) strength, and the five‐times sit‐to‐stand (5×STS) test [21, 22]—capture the capacity to perform physical tasks. We hypothesized that age‐group differences would be greater in functional indicators than in structural indicators.

## 2. Methods

### 2.1. Study Design

This community‐based cross‐sectional study examined body composition, anthropometric characteristics, and muscle function across age groups among women aged 50–89 years residing in rural areas of Asan, Korea. Data collection was conducted between March and November 2023.

### 2.2. Participants

Participants were community‐dwelling women aged 50–89 years (*n* = 211) recruited through convenience sampling from rural areas of Asan, Korea. Recruitment was conducted in 2023 through local health centers, community bulletin boards, and village public address announcements. Participants were not formally prescreened for specific diseases or functional status before enrollment. Individuals were eligible if they provided written informed consent after receiving an explanation of the study procedures, and those with severe musculoskeletal or neurological conditions that could interfere with safe or valid test performance (e.g., fracture within the past 12 months, advanced osteoarthritis, stroke, or Parkinson’s disease) were excluded before testing. A total of 228 participants were initially recruited. Of these, 17 were excluded due to these conditions, resulting in a final sample of 211 participants.

No monetary compensation was provided for participation. No a priori sample size calculation was performed, as this was a community‐based field investigation that included all eligible participants during the recruitment period.

Participants were stratified into four age groups: 50–59 years (*n* = 51), 60–69 years (*n* = 48), 70–79 years (*n* = 52), and 80–89 years (*n* = 60). Descriptive characteristics of each age group are presented in Table 1.

**TABLE 1:** Physical and body composition characteristics by age group.

Variable	50–59	60–69	70–79	80–89	*F*	*p*	*η* *p* ^2^
	(*n* = 51)	(*n* = 48)	(*n* = 52)	(*n* = 60)			
Age (yr)	55.1 ± 2.95	65.0 ± 3.09	75.5 ± 2.80	84.0 ± 2.67	—	—	—
Height (cm)	154.7 ± 4.62^a^	153.9 ± 5.22^a^	148.2 ± 5.50^b^	145.5 ± 5.52^c^	14.51	< 0.001	0.174
Weight (kg)	60.6 ± 8.41^a^	60.4 ± 8.94^a^	55.1 ± 7.49^b^	51.5 ± 8.89^c^	14.88	< 0.001	0.177
BMI (kg/m^2^)	24.8 ± 3.48	25.5 ± 3.30	25.1 ± 3.36	24.5 ± 4.13	0.78	0.506	0.011
Body fat (%)	33.5 ± 6.29^a^	36.5 ± 5.80^b^	38.3 ± 5.21^c^	38.0 ± 6.70^c^	7.04	< 0.001	0.093
SMM (kg)	21.6 ± 2.69^a^	20.2 ± 2.51^b^	17.4 ± 1.86^c^	16.2 ± 2.17^d^	60.57	< 0.001	0.467
SMI (kg/m^2^)	6.5 ± 0.68^a^	6.3 ± 0.62^a^	5.7 ± 0.58^b^	5.3 ± 0.65^c^	44.64	< 0.001	0.393

*Note:* Values are presented as mean ± SD. SMM, skeletal muscle mass; SMI, skeletal muscle index. Differences among age groups were examined using one‐way analysis of variance (ANOVA). Post hoc comparisons were performed using Tukey′s honestly significant difference test or the Games–Howell test, as appropriate, based on Levene′s test for homogeneity of variance. For each variable, superscript letters (a–d) indicate the results of post hoc comparisons among age groups: groups sharing the same superscript letter are not significantly different, whereas groups with different superscript letters differ significantly (*p* < 0.05). Letter assignments are independent across variables (rows).

Abbreviations: SMI, skeletal muscle index; SMM, skeletal muscle mass.

Information on menopausal status, including age at menopause and whether menopause was spontaneous or induced, was not collected in this study.

### 2.3. Study Procedure

The study followed a standardized protocol including recruitment and informed consent, body composition and anthropometric measurements, and muscle function assessments. After providing written consent, participants underwent anthropometric measurements (height, weight, thigh circumference [TC], and calf circumference [CF]), followed by assessment of HG strength, isometric KE strength, and the 5×STS test.

All participants completed the assessments in the same measurement order. All measurements were conducted by trained assessors who were instructed in the standardized protocols before data collection. All measurements were performed indoors at local community centers or senior welfare facilities in rural areas of Asan, Korea, under standardized conditions to ensure consistency across participants. Participants underwent a standardized rest period of approximately 10 min in a supine position before body composition assessment. Structural measurements, including body composition and anthropometric measures, took approximately 10 min. Functional measurements, including HG strength, isometric KE strength, and the 5×STS test, took approximately 15 min. The total time required to complete all assessments was approximately 30–40 min per participant.

### 2.4. Measurements

#### 2.4.1. Body Composition

Height and body weight were measured using an automated stadiometer–scale (BSM 330, InBody, Seoul, Korea). Body composition was assessed using a segmental multifrequency bioelectrical impedance analyzer (BWA 2.0, InBody, Seoul, Korea).

Participants rested in a supine position for 10 min before measurement. Measurements were conducted in the morning, approximately 3‐4 h after breakfast, and were not performed immediately after meals, based on participant self‐report. Participants were also asked to avoid strenuous physical activity before testing. During measurement, the arms were slightly abducted (∼15°) to avoid contact with the trunk, and electrodes were attached to both wrists and ankles according to the manufacturer’s guidelines.

Body mass index (BMI), SMM, fat mass, and percent body fat were obtained from the device output. The SMI was calculated as appendicular SMM (kg) divided by height squared (m^2^).

#### 2.4.2. Anthropometric Measurements

Circumference measurements were obtained using a nonelastic measuring tape (Hoechstmass, Germany) according to standardized anthropometric procedures [23]. Measurements were performed with participants standing in a relaxed posture, with the tape placed in contact with the skin without compressing the underlying soft tissue. TC was measured horizontally just below the gluteal fold, with the feet positioned slightly apart. CF was measured at the point of maximal girth between the knee and ankle.

Measurements were performed on both sides using the same protocol. Waist and hip circumferences were measured, and the waist‐to‐hip ratio (WHR) was calculated as waist circumference divided by hip circumference.

#### 2.4.3. Muscle Function

Muscle function was evaluated using HG strength, isometric KE strength, and the 5×STS test, which reflect upper‐limb strength, lower‐limb strength, and functional performance, respectively.

HG Strength: HG strength was measured using a digital dynamometer (TKK‐5401, TAKEI, Japan). Participants stood upright with their arms fully extended at their sides, with no contact between the arms and the trunk. Two trials were performed for each hand, and the highest value was recorded to the nearest 0.1 kg. HG is a reliable and widely used measure of overall muscle strength in older adults [17, 18].

#### 2.4.4. Isometric KE Strength

KE was measured using a dynamometer (TKK‐5710m, TAKEI, Japan) at approximately 60° of knee flexion according to the protocol described by Sipilä et al. [24]. Participants were seated with the trunk secured by a stabilization strap, and an ankle strap was fastened to the distal leg and connected to the dynamometer. Participants were instructed to perform a maximal voluntary isometric contraction for approximately 3 s. Two trials were performed for each leg, and the highest value was recorded to the nearest 0.1 kg. Both dynamometers provide peak force values displayed digitally, and these values were used for analysis. Measurements were based on peak force output rather than continuous signal recording.

#### 2.4.5. 5×STS Test

5×STS was administered as a performance‐based assessment of integrated lower‐extremity function. The test has been widely used to assess lower‐extremity strength and functional performance in older adults [21, 22]. Participants began in a seated position on a chair with a backrest, with their arms crossed over the chest, and were instructed to stand and sit five times as quickly as possible after the start signal. The total completion time was recorded in seconds using a stopwatch (Casio, Japan). The test has demonstrated high test–retest reliability in adult populations [25].

### 2.5. Statistical Analysis

Statistical analyses were performed using IBM SPSS Statistics Version 21.0 (IBM Corp., Armonk, NY, USA). Descriptive statistics are presented as mean ± standard deviation (SD).

Between‐group differences were examined using one‐way analysis of variance (ANOVA) followed by post hoc pairwise comparisons. Homogeneity of variance was assessed using Levene’s test, and normality was evaluated via the Shapiro–Wilk test. Tukey’s honestly significant difference test was applied when homogeneity assumptions were met, whereas the Games–Howell procedure was used when variances were unequal. Statistical significance was set at a two‐tailed *α* level of 0.05.

To compare age‐related patterns of structural and functional indicators, relative indices were calculated by normalizing SMI, SMM, TC, CF, HG, KE, and 5×STS values to the mean of the 50–59 age group (=100), consistent with prior age‐stratified studies using similar normalization approaches to compare relative performance across age groups [13]. This normalization facilitated intuitive comparison of relative magnitudes of change while eliminating unit differences across variables.

A mixed‐design repeated‐measures ANOVA was conducted with age group (four levels) as the between‐subject factor and domain score (structural vs. functional) as the within‐subject factor. Both structural and functional domains were assessed at a single time point. Because each participant contributed values for both structural and functional domains, the domain was treated as a within‐subject factor, allowing direct comparison of domain‐specific differences within individuals. Effect sizes were reported as partial eta squared (*η* *p* ^2^) to quantify the magnitude of age‐related differences and to complement *p*‐values by providing information on the practical significance of observed effects. Effect sizes were interpreted as small (*η* *p* ^2^ ≥ 0.01), medium (*η* *p* ^2^ ≥ 0.06), and large (*η* *p* ^2^ ≥ 0.14). The sphericity assumption was evaluated using Mauchly’s test, and the Greenhouse–Geisser correction was applied when the assumption was violated. Normality was assessed using the Shapiro–Wilk test, and independence of observations was ensured by the between‐subject design.

The structural domain included SMI, SMM, TC, and CF, whereas the functional domain comprised HG, KE, and 5×STS, consistent with prior research distinguishing muscle quantity from functional capacity [3, 21]. Domain scores were calculated by averaging the relative indices within each domain. For bilaterally measured variables—including TC, CF, HG, and KE—the mean of the left and right sides was used as the representative value. Although minor differences between sides were observed, no consistent pattern of asymmetry was identified. To ensure directional consistency, 5×STS time (seconds) was inverse‐transformed (1/time), with higher values indicating better performance before calculating the relative indices. Age was analyzed as a categorical variable using four decade‐based groups to facilitate age‐stratified comparison. All analyzed variables had complete data in the final sample of 211 participants.

An analysis of covariance (ANCOVA) was conducted for 5×STS and KE to examine whether age‐related differences in functional indicators were independent of body composition, using body fat percentage as a covariate. Body fat percentage was selected because it reflects overall body composition status independently of muscle quantity and is closely associated with functional performance, whereas other body composition variables (e.g., SMM, SMI, body weight, and BMI) were not included to avoid conceptual overlap with the outcome measures and potential over‐adjustment bias.

Normalization was performed using the 50–59 age group as the reference to allow direct comparison of age‐related differences across groups.

## 3. Results

### 3.1. Physical and Body Composition Characteristics

Physical and body composition characteristics differed significantly across age groups (Table 1).

Height, body weight, SMM, and SMI were progressively lower in older age groups (all *p* < 0.001). The mean SMI declined from 6.5 ± 0.68 kg/m^2^ in the 50–59 group to 5.3 ± 0.65 kg/m^2^ in the 80–89 group. Percent body fat increased significantly across age groups (*p* < 0.001), whereas BMI did not differ significantly (*p* = 0.506).

### 3.2. Morphological Characteristics

TC and CF both were significantly lower in older age groups (*p* < 0.001) (Table 2).

**TABLE 2:** Morphological characteristics by age group.

Variable		50–59	60–69	70–79	80–89	*F*	*p*	*η* *p* ^2^
		(*n* = 51)	(*n* = 48)	(*n* = 52)	(*n* = 60)			
TC (cm)	R	53.0 ± 3.99^a^	50.3 ± 4.53^b^	48.0 ± 4.88^c^	44.9 ± 4.01^d^	34.60	< 0.001	0.334
	L	52.6 ± 3.97^a^	50.3 ± 4.89^b^	46.8 ± 5.66^c^	44.6 ± 4.09^d^	31.18	< 0.001	0.311

CF (cm)	R	35.3 ± 2.59^a^	34.7 ± 5.21^ab^	32.3 ± 2.63^b^	30.8 ± 2.54^c^	20.80	< 0.001	0.232
	L	35.3 ± 2.66^a^	34.3 ± 2.77^ab^	32.0 ± 2.79^b^	30.8 ± 2.19^c^	34.80	< 0.001	0.335

Waist–hip ratio		0.87 ± 0.083^a^	0.90 ± 0.053^b^	0.91 ± 0.067^b^	0.91 ± 0.087^b^	3.89	0.010	0.053

*Note:* Values are presented as mean ± SD. TC, thigh circumference; CF, calf circumference. Post hoc comparisons were performed using Tukey′s honestly significant difference test or the Games–Howell test, as appropriate, based on Levene′s test for homogeneity of variance. For each variable, superscript letters (a–d) indicate the results of post hoc comparisons among age groups: groups sharing the same superscript letter are not significantly different, whereas groups with different superscript letters differ significantly (*p* < 0.05). Letter assignments are independent across variables (rows).

Abbreviations: CF, calf circumference; TC, thigh circumference.

TC showed a consistent stepwise pattern across successive decades, while CF demonstrated more pronounced differences beginning in the 70–79 age group. WHR also increased significantly across age groups (*p* = 0.010).

### 3.3. Muscle Function

All muscle function variables differed significantly across age groups (all *p* < 0.001) (Table 3).

**TABLE 3:** Muscle function by age group.

Variable		50–59	60–69	70–79	80–89	*F*	*p*	*η* *p* ^2^
		(*n* = 51)	(*n* = 48)	(*n* = 52)	(*n* = 60)			
HG (kg)	R	24.5 ± 5.24^a^	22.5 ± 4.46^b^	17.0 ± 4.46^c^	14.2 ± 4.60^d^	56.28	< 0.001	0.449
	L	24.0 ± 4.88^a^	21.9 ± 3.83^b^	18.5 ± 4.20^c^	14.5 ± 3.74^d^	54.71	< 0.001	0.442

KE (kg)	R	40.1 ± 10.72^a^	27.7 ± 8.28^b^	18.6 ± 7.65^c^	15.3 ± 6.09^c^	96.63	< 0.001	0.583
	L	40.2 ± 9.19^a^	27.7 ± 8.15^b^	19.6 ± 6.10^c^	14.3 ± 5.91^d^	125.14	< 0.001	0.645

5×STS time (s)		7.0 ± 1.75^a^	9.8 ± 4.02^b^	12.3 ± 4.25^c^	15.2 ± 6.60^d^	31.63	< 0.001	0.314

*Note:* Values are presented as mean ± SD. HG, handgrip strength; KE, isometric knee extensor strength; 5×STS, five‐times sit‐to‐stand test. Post hoc comparisons were performed using Tukey′s honestly significant difference test or the Games–Howell test, as appropriate. For each variable, superscript letters (a–d) indicate the results of post hoc comparisons among age groups: groups sharing the same superscript letter are not significantly different, whereas groups with different superscript letters differ significantly (*p* < 0.05). Letter assignments are independent across variables (rows).

HG and KE were progressively lower in older age groups, with the largest effect sizes observed for KE (partial *η* *p* ^2^ = 0.583–0.645). In contrast, 5×STS completion time was substantially longer in older age groups, indicating reduced functional performance in older participants.

### 3.4. Integrated Pattern of Structural and Functional Changes

**FIGURE 1:**
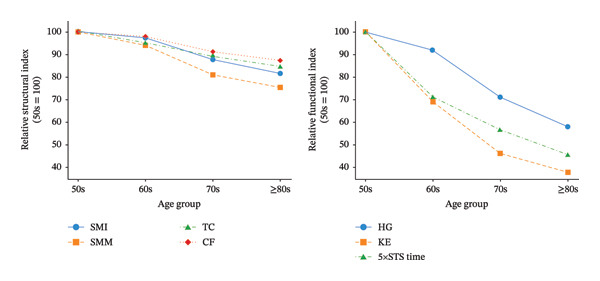
Divergent patterns of structural and functional muscle indices across age groups, normalized to the 50–59 age group (=100). Statistical significance for each variable is reported in Tables 1, 2, and 3.

To facilitate comparison across indicators, relative indices were calculated by normalizing each variable to the mean of the 50–59 age group (=100) (Table 4; Figure 1).

**TABLE 4:** Relative structural and functional indices normalized to the 50–59 age group (=100) across age groups.

Domain	Relative index	50–59	60–69	70–79	80–89	*p*	*η* *p* ^2^
		(*n* = 51)	(*n* = 48)	(*n* = 52)	(*n* = 60)		
Structural	Relative SMI	100	96.9	87.7	81.5	< 0.001	0.393
	Relative SMM	100	93.8	80.9	75.3	< 0.001	0.467
	Relative TC	100	95.2	89.8	84.7	< 0.001	0.334
	Relative CF	100	97.6	91.2	87.2	< 0.001	0.335

Functional	Relative HG	100	91.8	71.4	58.0	< 0.001	0.446
	Relative KE	100	69.1	46.4	38.1	< 0.001	0.614
	Relative 5×STS performance^†^	100	71.4	56.9	46.0	< 0.001	0.314

*Note:* *p* and *η* *p* ^2^ values are based on one‐way ANOVA and correspond to the statistical comparisons reported in Tables 1, 2, and 3. HG, handgrip strength; KE, isometric knee extensor strength; 5×STS, five‐times sit‐to‐stand test.

Abbreviations: CF, calf circumference; SMI, skeletal muscle index; SMM, skeletal muscle mass; TC, thigh circumference.

^†^5×STS time was reciprocally transformed (1/time) so that higher values indicate better performance.

Statistical significance and effect sizes are summarized in Table 4, with detailed statistical results reported in Tables 1, 2, and 3. Structural indices (SMI, SMM, TC, and CF) were lower in older age groups. Functional indices (HG, KE, and reciprocal‐transformed 5×STS) showed proportionally larger differences between age groups, particularly in the 70–79 and 80–89 groups.

For directional consistency, 5×STS time was reciprocal‐transformed (1/time) before normalization so that higher values reflected better performance.

### 3.5. Structural and Functional Domain Differences

Mixed‐design ANOVA demonstrated a significant domain × age group interaction (*F* [3, 207] = 67.98, *p* < 0.001, partial *η* *p* ^2^ = 0.496), indicating that age‐related patterns differed between structural and functional domains, with a greater relative magnitude of change in the functional domain. The interaction effect was large in magnitude, and functional indicators, particularly KE (*η* *p* ^2^ = 0.583–0.645), showed larger effect sizes than SMI (*η* *p* ^2^ = 0.393). Significant main effects were also observed for domain (*F* [1, 207] = 478.67, *p* < 0.001, partial *η* *p* ^2^ = 0.698) and age group (*F* [3, 207] = 175.55, *p* < 0.001, partial *η* *p* ^2^ = 0.718). To further examine whether age‐related differences in functional indicators were independent of body composition, an ANCOVA was conducted with body fat percentage included as a covariate (Table 5).

**TABLE 5:** ANCOVA results for functional indicators after adjustment for body fat percentage.

Outcome	Source	df	*F*	*p*	*η* *p* ^2^
5×STS	Body fat (%)	1	1.148	0.285	0.006
	Age group	3	27.554	< 0.001	0.286

KE	Body fat (%)	1	6.218	0.013	0.029
	Age group	3	104.052	< 0.001	0.602

*Note:* Body fat percentage was included as a covariate.

Abbreviations: KE (kg), isometric knee extension strength; 5×STS (s), five‐times sit‐to‐stand test.

The effects of age group remained statistically significant after adjustment for both 5×STS (*F* = 27.554, *p* < 0.001, *η* *p* ^2^ = 0.286) and KE (*F* = 104.052, *p* < 0.001, *η* *p* ^2^ = 0.602). Body fat percentage was significantly associated with KE (*p* = 0.013) but not with 5×STS (*p* = 0.285), suggesting that age‐group differences in functional indicators persisted after accounting for body fat percentage.

## 4. Discussion

### 4.1. Structural–Functional Divergence in Age‐Related Muscle Changes

This study demonstrates a clear divergence between structural and functional muscle characteristics across age groups in rural community‐dwelling women. These findings indicate that structural measures alone may underestimate the magnitude of functional differences between groups. This pattern suggests that functional vulnerability in later life may be underestimated when assessment relies predominantly on structural indicators. This limitation may be particularly relevant in populations with limited access to comprehensive diagnostic evaluation, where functional indicators may provide a more sensitive indication of age‐related decline.

This structural–functional dissociation indicates that age‐related functional decline cannot be explained solely by reductions in muscle quantity. Circumference‐based indicators, such as TC and CF, may also reflect subcutaneous adipose tissue and other noncontractile components and should therefore be interpreted as indirect indicators of regional limb size rather than isolated measures of muscle quantity. Although physiological determinants were not directly assessed, prior research suggests that age‐related neuromuscular and motor control factors may partly contribute to the larger differences observed in functional indicators relative to structural indicators [11, 12]. Lower‐extremity strength, particularly KE strength, plays a critical role in key mobility tasks, such as walking, standing, and chair rising [16, 24]. Performance‐based measures reflect integrated functional capacity that cannot be captured by a single strength metric. They are also associated with functional decline and adverse health outcomes, underscoring their clinical relevance in aging populations [26]. Although HG strength is widely used as a practical biomarker of overall muscle function, evidence suggests that it may not fully represent lower‐limb strength or real‐world performance capacity [27]. The ANCOVA results further support this interpretation, showing that age‐related differences in functional indicators persisted after adjustment for body fat percentage. Effect sizes were considered alongside *p*‐values to contextualize the magnitude and practical relevance of age‐related differences in functional indicators. These results suggest that age‐related differences in functional capacity are not fully explained by body composition alone. This pattern is consistent with evidence indicating that rural community‐dwelling older adults may be at elevated risk of functional decline, partly due to limited access to healthcare resources, lower engagement in structured exercise, and distinct lifestyle patterns [20]. These contextual factors may further explain the greater age‐related differences observed in functional indicators in this study.

Potentially important confounding factors—including physical activity, nutritional status, chronic disease burden, and menopausal status—were not incorporated into the analysis. These factors may have contributed to the observed structural–functional divergence. In particular, lower levels of physical activity and a higher burden of chronic conditions could disproportionately affect functional performance, potentially explaining the greater decline observed in functional indicators relative to structural indicators [28]. Variations in nutritional status and hormonal changes associated with menopause may have also influenced both muscle mass and function [29].

### 4.2. Differential Magnitude of Structural and Functional Decline

A clear divergence in age‐related patterns was observed between structural and functional domains, with performance‐based indicators showing larger differences between age groups. In the 80–89 age group, the SMI relative index remained at approximately 80% of the values observed in the 50–59 age group, whereas functional indices were approximately half of those reference values, illustrating a pronounced structural–functional divergence in the oldest age group. This contrast emphasizes the importance of integrating structural and performance‐based measures to better characterize age‐group differences in muscle function.

Although SMM was progressively lower in older age groups, differences in 5×STS performance were markedly larger in the oldest groups. This pattern demonstrates that the magnitude of age‐group differences in functional indicators differs across age groups rather than progressing uniformly. The 5×STS test reflects not only lower‐limb strength but also balance, coordination, and movement strategy during repeated sit‐to‐stand transitions [22, 30]. These multidimensional demands may help explain the larger differences observed in 5×STS performance in the oldest groups. As direct neuromuscular or biomechanical measurements were not performed, caution is warranted when attributing these differences to specific physiological mechanisms.

Previous studies have similarly reported differences between structural and functional indicators of aging. Choe et al. [31] suggested that muscle mass influences frailty indirectly through functional capacity, while Asano et al. [32] observed that reductions in physical performance may exceed those observed in muscle mass with advancing age. Age‐related differences in 5×STS performance have been documented across diverse populations, including community‐dwelling older adults in China [33] and a pooled international sample spanning 14 countries [34]. Reference values for HG strength have similarly demonstrated marked age‐group differences in older adults from Spain and Latin America [18]. These findings extend this evidence by demonstrating that structural and functional patterns may diverge across age groups, highlighting the importance of evaluating these domains separately.

Consistent with this interpretation, functional indicators demonstrated larger between‐group differences than structural indicators in this study. This dissociation between muscle structure and performance supports the inclusion of function‐focused assessments when characterizing age‐group differences in functional performance.

### 4.3. Age‐Stratified Patterns of Functional Decline

A notable finding was the larger differences in 5×STS performance observed in the older age groups, indicating greater differences in functional performance in the later decades. Similar age‐dependent patterns have been reported, with performance measures showing greater declines at advanced ages [33, 34], and mobility‐related indicators, such as gait speed and HG strength, also demonstrating steeper declines later in life [1, 6]. Notably, similar trends have been observed in community‐dwelling older adults in rural settings, where limited access to healthcare resources and lower engagement in structured physical activity may further accelerate functional decline [20].

In this context, age‐related functional differences are better interpreted by considering not only between‐group differences but also the magnitude of differences across age groups. Taken together, these results highlight the particular sensitivity of performance‐based indicators in detecting age‐related functional differences across defined age groups.

This study has several limitations that should be acknowledged. First, the cross‐sectional design precludes causal inference regarding differences in muscle structure and function across age groups. Second, participants were recruited using convenience sampling from a single rural community, which may introduce selection bias and limit generalizability to urban populations or to men. Furthermore, as participation was voluntary, individuals with severe functional limitations may have been less likely to enroll, potentially resulting in healthy volunteer bias. In addition, the oldest age group (80–89 years) may have been subject to survivorship bias, potentially leading to underestimation of age‐group differences in the oldest group. Third, several potentially important confounding factors were not included in the analysis, including sex‐specific factors, such as menopausal status, occupational characteristics, and medication use, which may have influenced the observed differences in structural and functional indicators. Furthermore, musculoskeletal pain and rural occupational demands, including prolonged agricultural work, were not accounted for in this study and may have disproportionately affected functional performance. In addition, the absence of strict fasting conditions before BIA assessment may have introduced variability in body composition estimates, as BIA measurements can be influenced by hydration status and recent food intake. The dynamometers used in this study assessed peak force output but did not evaluate rate of force development (RFD), which may provide additional information on muscle function in older adults. In addition, formal intra‐ or inter‐rater reliability analyses for anthropometric and strength assessments were not performed in this study, although all measurements were conducted by trained assessors using standardized protocols. Future longitudinal studies with geographically diverse samples and comprehensive health assessments will be important for clarifying the trajectories and determinants of structural and functional aging.

## 5. Conclusions

These findings demonstrate that structural and functional muscle indicators follow divergent age‐related patterns in rural community‐dwelling women. While structural indicators showed smaller differences across age groups, performance‐based functional indicators showed substantially larger age‐group differences. These findings suggest that age‐group differences across muscle indicators are not uniform.

Together, these results support the integration of structural and functional assessments and highlight the value of incorporating functional assessment into age‐stratified evaluation approaches in rural settings. Such approaches may help characterize age‐group differences in functional performance and inform community‐based health management strategies, particularly in resource‐limited rural populations.

From a practical perspective, functional performance assessments should be incorporated alongside structural measures in community and clinical settings. For women in their 60s and 70s, simple tools, such as 5×STS, may help characterize functional performance differences across age groups, while targeted exercise interventions focusing on lower‐extremity strength are particularly warranted for those in their 80s. Health professionals and exercise practitioners in rural settings—including local health centers and senior welfare facilities—can benefit from incorporating such assessments into age‐stratified functional evaluation. Because this study was cross‐sectional and did not assess prospective clinical outcomes, longitudinal studies are needed to further clarify the clinical utility of these assessments.

## Author Contributions

Jaeyong Park: conceptualization, methodology, formal analysis, and writing–original draft. Youngju Song: investigation, data curation, and writing–review and editing.

## Funding

This research received no specific grant from any funding agency in the public, commercial, or not‐for‐profit sectors.

## Disclosure

All authors approved the final version of the manuscript.

## Ethics Statement

The study protocol was approved by the Institutional Review Board of Sunmoon University (SM‐202212‐051‐2). All procedures were conducted in accordance with the Declaration of Helsinki, and written informed consent was obtained from all participants before participation.

## Conflicts of Interest

The authors declare no conflicts of interest.

## Data Availability

The datasets generated and/or analyzed during this study are available from the corresponding author upon reasonable request.
